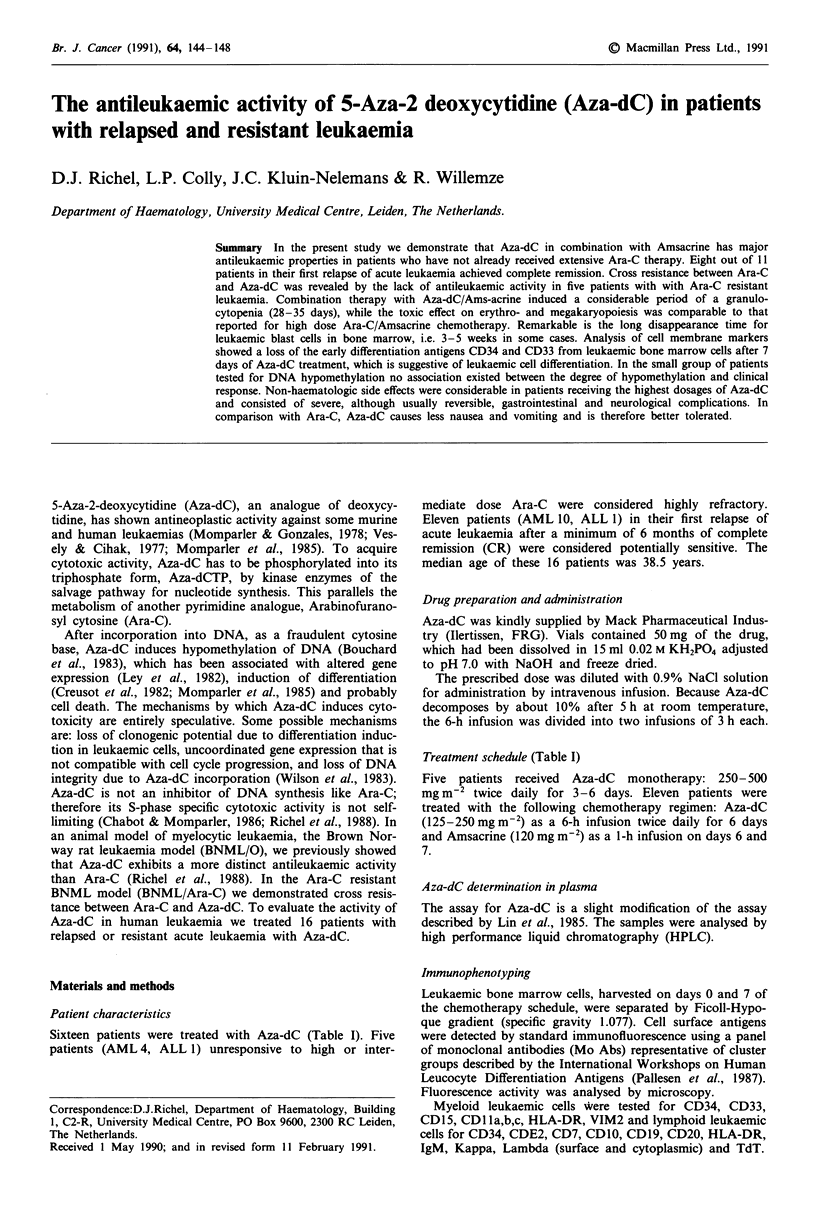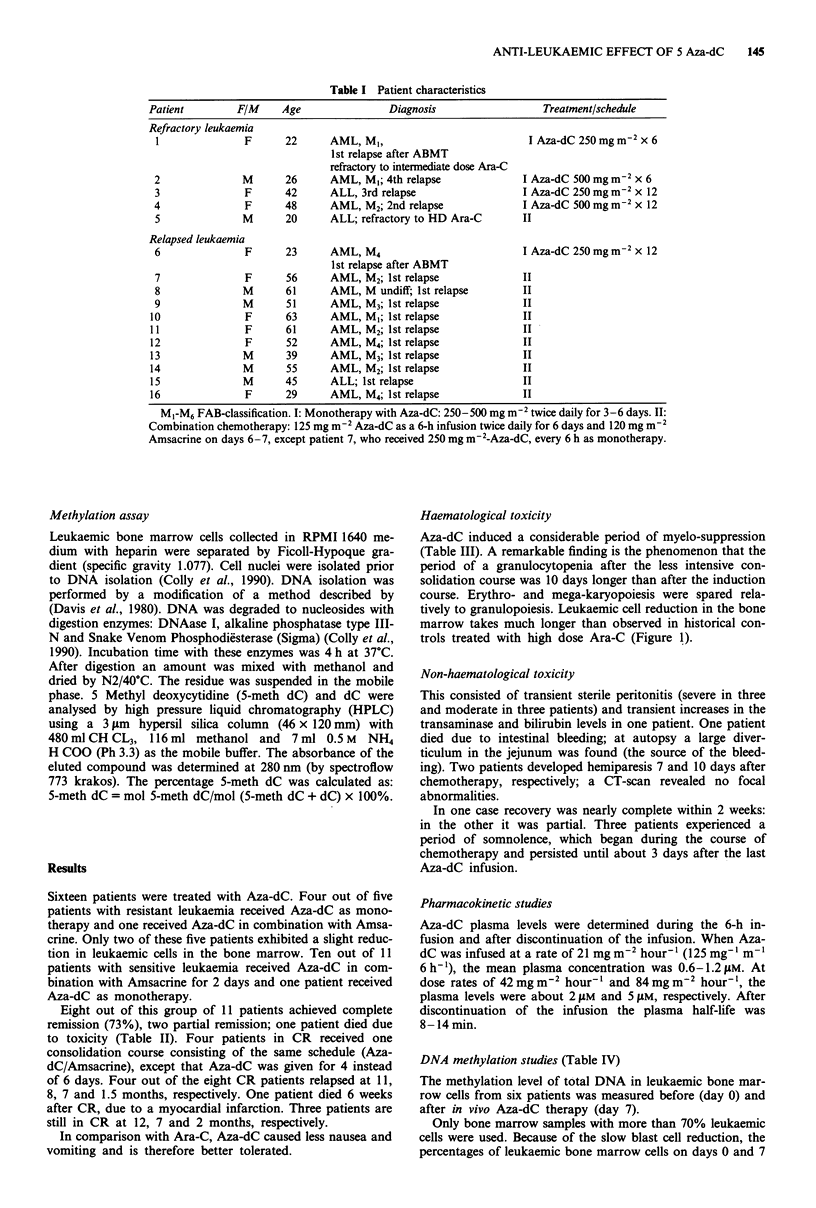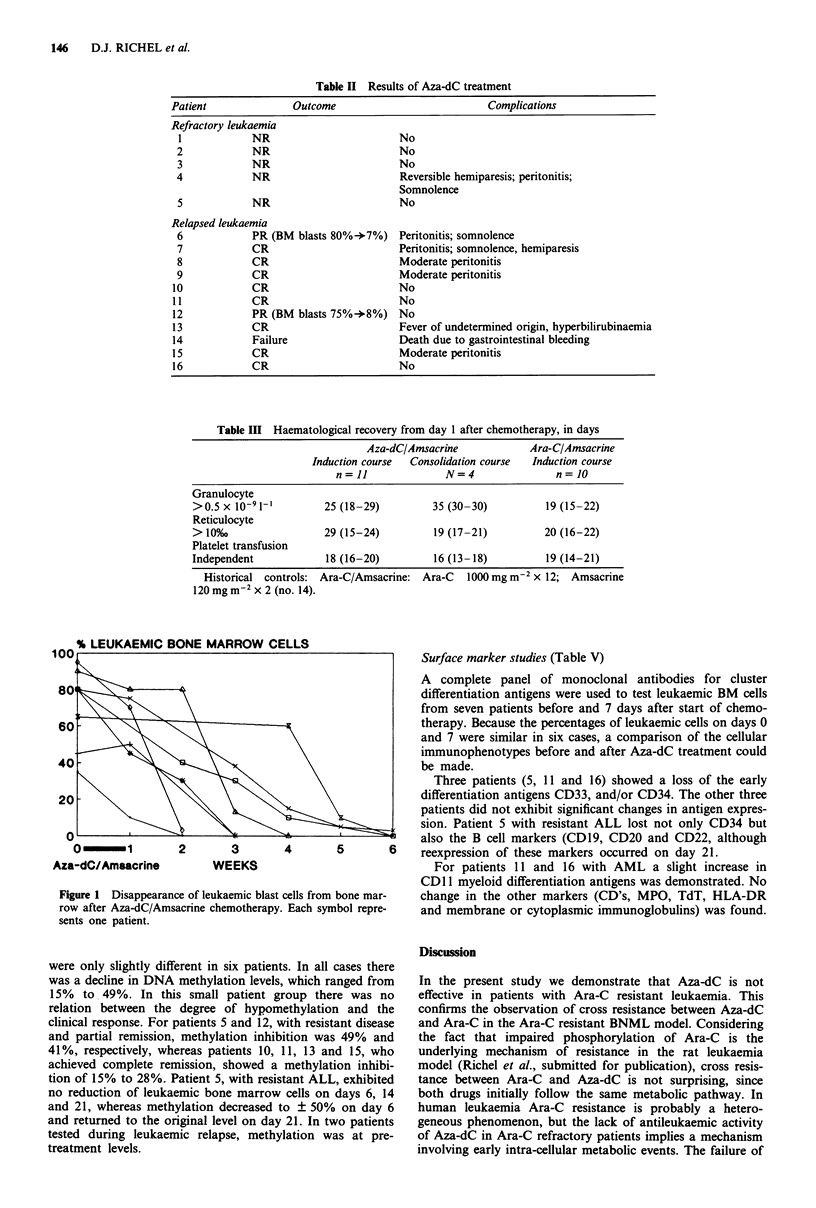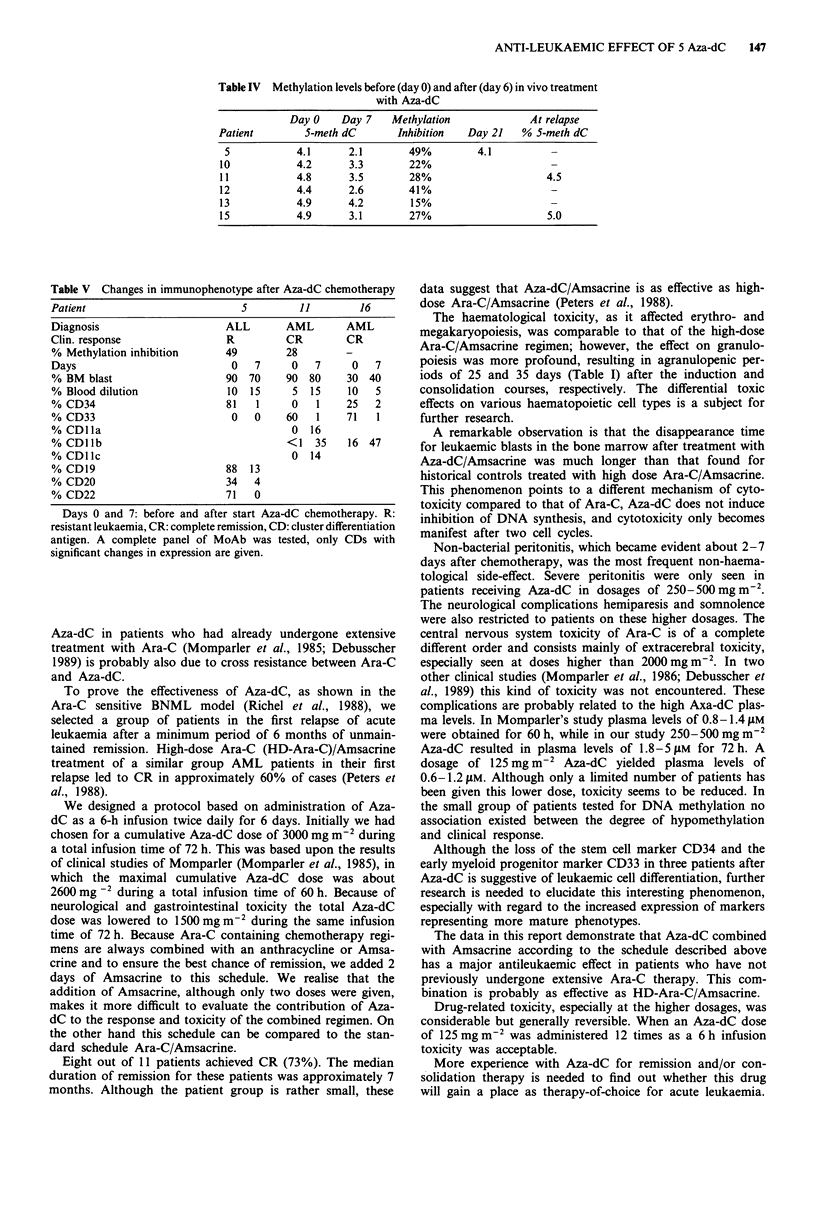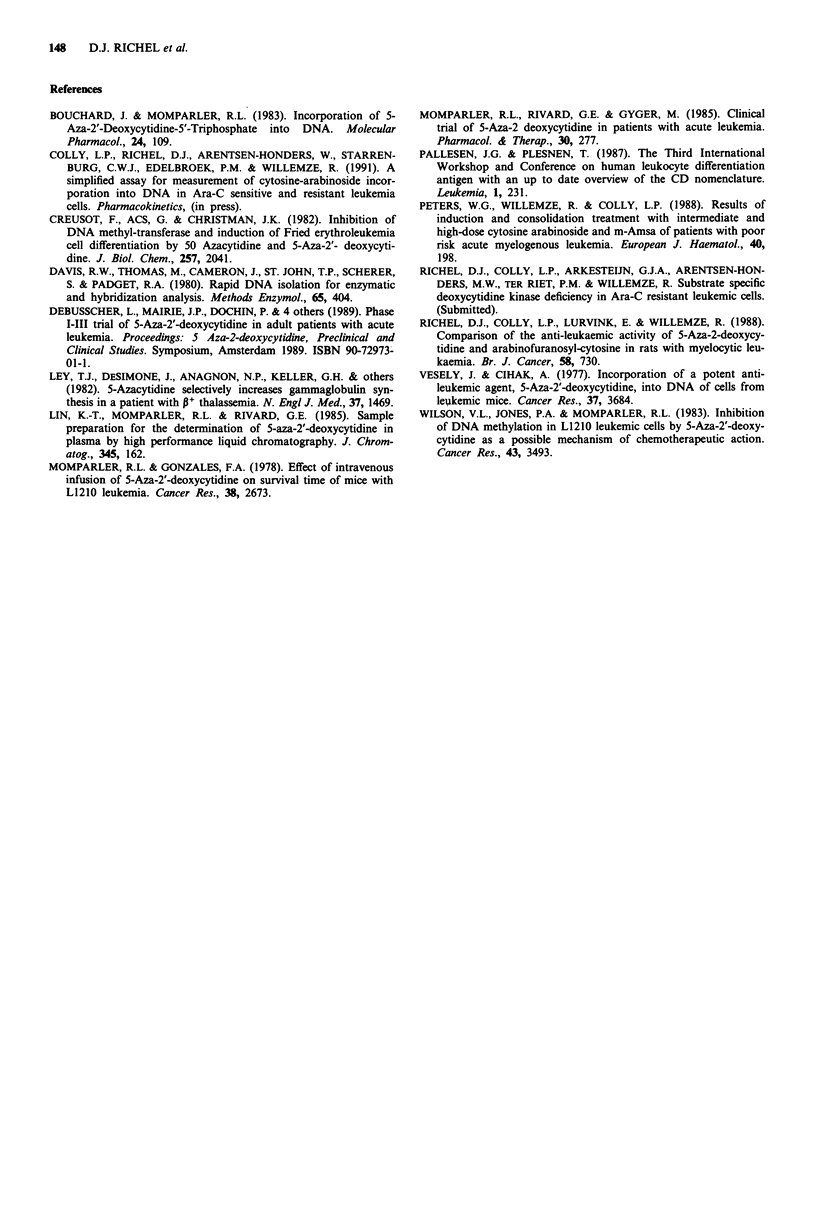# The antileukaemic activity of 5-Aza-2 deoxycytidine (Aza-dC) in patients with relapsed and resistant leukaemia.

**DOI:** 10.1038/bjc.1991.258

**Published:** 1991-07

**Authors:** D. J. Richel, L. P. Colly, J. C. Kluin-Nelemans, R. Willemze

**Affiliations:** Department of Haematology, University Medical Centre, Leiden, The Netherlands.

## Abstract

In the present study we demonstrate that Aza-dC in combination with Amsacrine has major antileukaemic properties in patients who have not already received extensive Ara-C therapy. Eight out of 11 patients in their first relapse of acute leukaemia achieved complete remission. Cross resistance between Ara-C and Aza-dC was revealed by the lack of antileukaemic activity in five patients with with Ara-C resistant leukaemia. Combination therapy with Aza-dC/Ams-acrine induced a considerable period of a granulocytopenia (28-35 days), while the toxic effect on erythro- and megakaryopoiesis was comparable to that reported for high dose Ara-C/Amsacrine chemotherapy. Remarkable is the long disappearance time for leukaemic blast cells in bone marrow, i.e. 3-5 weeks in some cases. Analysis of cell membrane markers showed a loss of the early differentiation antigens CD34 and CD33 from leukaemic bone marrow cells after 7 days of Aza-dC treatment, which is suggestive of leukaemic cell differentiation. In the small group of patients tested for DNA hypomethylation no association existed between the degree of hypomethylation and clinical response. Non-haematologic side effects were considerable in patients receiving the highest dosages of Aza-dC and consisted of severe, although usually reversible, gastrointestinal and neurological complications. In comparison with Ara-C, Aza-dC causes less nausea and vomiting and is therefore better tolerated.


### The antileukaemic activity of 5-Aza-2 deoxycytidine (Aza-dC) in patients with relapsed and resistant leukaemia

D.J. Richel, L.P. Colly, J.C. Kluin-Nelemans & R. Willemze

*Department of Haematology, University Medical Centre, Leiden, The Netherlands.*

**Summary** In the present study we demonstrate that Aza-dC in combination with Amsacrine has major antileukaemic properties in patients who have not already received extensive Ara-C therapy. Eight out of 11 patients in their first relapse of acute leukaemia achieved complete remission. Cross resistance between Ara-C and Aza-dC was revealed by the lack of antileukaemic activity in five patients with with Ara-C resistant leukaemia. Combination therapy with Aza-dC/Ams-acrine induced a considerable period of a granulocytopenia (28–35 days), while the toxic effect on erythro- and megakaryopoiesis was comparable to that reported for high dose Ara-C/Amsacrine chemotherapy. Remarkable is the long disappearance time for leukaemic blast cells in bone marrow, i.e. 3–5 weeks in some cases. Analysis of cell membrane markers showed a loss of the early differentiation antigens CD34 and CD33 from leukaemic bone marrow cells after 7 days of Aza-dC treatment, which is suggestive of leukaemic cell differentiation. In the small group of patients tested for DNA hypomethylation no association existed between the degree of hypomethylation and clinical response. Non-haematologic side effects were considerable in patients receiving the highest dosages of Aza-dC and consisted of severe, although usually reversible, gastrointestinal and neurological complications. In comparison with Ara-C, Aza-dC causes less nausea and vomiting and is therefore better tolerated.

5-Aza-2-deoxycytidine (Aza-dC), an analogue of deoxycytidine, has shown antineoplastic activity against some murine and human leukaemias (Momparler & Gonzales, 1978; Vesely & Cihak, 1977; Momparler *et al.*, 1985). To acquire cytotoxic activity, Aza-dC has to be phosphorylated into its triphosphate form, Aza-dCTP, by kinase enzymes of the salvage pathway for nucleotide synthesis. This parallels the metabolism of another pyrimidine analogue, Arabinofuranosyl cytosine (Ara-C).

After incorporation into DNA, as a fraudulent cytosine base, Aza-dC induces hypomethylation of DNA (Bouchard *et al.*, 1983), which has been associated with altered gene expression (Ley *et al.*, 1982), induction of differentiation (Creusot *et al.*, 1982; Momparler *et al.*, 1985) and probably cell death. The mechanisms by which Aza-dC induces cytotoxicity are entirely speculative. Some possible mechanisms are: loss of clonogenic potential due to differentiation induction in leukaemic cells, uncoordinated gene expression that is not compatible with cell cycle progression, and loss of DNA integrity due to Aza-dC incorporation (Wilson *et al.*, 1983). Aza-dC is not an inhibitor of DNA synthesis like Ara-C; therefore its S-phase specific cytotoxic activity is not self-limiting (Chabot & Momparler, 1986; Richel *et al.*, 1988). In an animal model of myelocytic leukaemia, the Brown Norway rat leukaemia model (BNML/O), we previously showed that Aza-dC exhibits a more distinct antileukaemic activity than Ara-C (Richel *et al.*, 1988). In the Ara-C resistant BNML model (BNML/Ara-C) we demonstrated cross resistance between Ara-C and Aza-dC. To evaluate the activity of Aza-dC in human leukaemia we treated 16 patients with relapsed or resistant acute leukaemia with Aza-dC.

### Materials and methods

### *Patient characteristics*

Sixteen patients were treated with Aza-dC (Table I). Five patients (AML 4, ALL 1) unresponsive to high or intermediate dose Ara-C were considered highly refractory. Eleven patients (AML 10, ALL 1) in their first relapse of acute leukaemia after a minimum of 6 months of complete remission (CR) were considered potentially sensitive. The median age of these 16 patients was 38.5 years.

### *Drug preparation and administration*

Aza-dC was kindly supplied by Mack Pharmaceutical Industry (Ilertissen, FRG). Vials contained 50 mg of the drug, which had been dissolved in 15 ml 0.02 M $KH_2PO_4$ adjusted to pH 7.0 with NaOH and freeze dried.

The prescribed dose was diluted with 0.9% NaCl solution for administration by intravenous infusion. Because Aza-dC decomposes by about 10% after 5 h at room temperature, the 6-h infusion was divided into two infusions of 3 h each.

### *Treatment schedule* (Table I)

Five patients received Aza-dC monotherapy: 250–500 mg $m^{-2}$ twice daily for 3–6 days. Eleven patients were treated with the following chemotherapy regimen: Aza-dC (125–250 mg $m^{-2}$) as a 6-h infusion twice daily for 6 days and Amsacrine (120 mg $m^{-2}$) as a 1-h infusion on days 6 and 7.

### *Aza-dC determination in plasma*

The assay for Aza-dC is a slight modification of the assay described by Lin *et al.*, 1985. The samples were analysed by high performance liquid chromatography (HPLC).

### *Immunophenotyping*

Leukaemic bone marrow cells, harvested on days 0 and 7 of the chemotherapy schedule, were separated by Ficoll-Hypoque gradient (specific gravity 1.077). Cell surface antigens were detected by standard immunofluorescence using a panel of monoclonal antibodies (Mo Abs) representative of cluster groups described by the International Workshops on Human Leucocyte Differentiation Antigens (Pallesen *et al.*, 1987). Fluorescence activity was analysed by microscopy.

Myeloid leukaemic cells were tested for CD34, CD33, CD15, CD11a,b,c, HLA-DR, VIM2 and lymphoid leukaemic cells for CD34, CDE2, CD7, CD10, CD19, CD20, HLA-DR, IgM, Kappa, Lambda (surface and cytoplasmic) and TdT.

Correspondence: D.J.Richel, Department of Haematology, Building 1, C2-R, University Medical Centre, PO Box 9600, 2300 RC Leiden, The Netherlands.
Received 1 May 1990; and in revised form 11 February 1991.

**Table I** Patient characteristics

| *Patient* | *F/M* | *Age* | *Diagnosis* | *Treatment/schedule* |
|---|---|---|---|---|
| *Refractory leukaemia* | | | | |
| 1 | F | 22 | AML, $M_1$, 1st relapse after ABMT refractory to intermediate dose Ara-C | I Aza-dC 250 mg $m^{-2}$ × 6 |
| 2 | M | 26 | AML, $M_1$; 4th relapse | I Aza-dC 500 mg $m^{-2}$ × 6 |
| 3 | F | 42 | ALL, 3rd relapse | I Aza-dC 250 mg $m^{-2}$ × 12 |
| 4 | F | 48 | AML, $M_2$; 2nd relapse | I Aza-dC 500 mg $m^{-2}$ × 12 |
| 5 | M | 20 | ALL; refractory to HD Ara-C | II |
| *Relapsed leukaemia* | | | | |
| 6 | F | 23 | AML, $M_4$ 1st relapse after ABMT | I Aza-dC 250 mg $m^{-2}$ × 12 |
| 7 | F | 56 | AML, $M_2$; 1st relapse | II |
| 8 | M | 61 | AML, M undiff; 1st relapse | II |
| 9 | M | 51 | AML, $M_3$; 1st relapse | II |
| 10 | F | 63 | AML, $M_1$; 1st relapse | II |
| 11 | F | 61 | AML, $M_2$; 1st relapse | II |
| 12 | F | 52 | AML, $M_4$; 1st relapse | II |
| 13 | M | 39 | AML, $M_3$; 1st relapse | II |
| 14 | M | 55 | AML, $M_2$; 1st relapse | II |
| 15 | M | 45 | ALL; 1st relapse | II |
| 16 | F | 29 | AML, $M_4$; 1st relapse | II |

$M_1$-$M_6$ FAB-classification. I: Monotherapy with Aza-dC: 250–500 mg $m^{-2}$ twice daily for 3–6 days. II: Combination chemotherapy: 125 mg $m^{-2}$ Aza-dC as a 6-h infusion twice daily for 6 days and 120 mg $m^{-2}$ Amsacrine on days 6–7, except patient 7, who received 250 mg $m^{-2}$-Aza-dC, every 6 h as monotherapy.

### *Methylation assay*

Leukaemic bone marrow cells collected in RPMI 1640 medium with heparin were separated by Ficoll-Hypoque gradient (specific gravity 1.077). Cell nuclei were isolated prior to DNA isolation (Colly *et al.*, 1990). DNA isolation was performed by a modification of a method described by (Davis *et al.*, 1980). DNA was degraded to nucleosides with digestion enzymes: DNAase I, alkaline phosphatase type III-N and Snake Venom Phosphodiësterase (Sigma) (Colly *et al.*, 1990). Incubation time with these enzymes was 4 h at 37°C. After digestion an amount was mixed with methanol and dried by N2/40°C. The residue was suspended in the mobile phase. 5 Methyl deoxycytidine (5-meth dC) and dC were analysed by high pressure liquid chromatography (HPLC) using a 3 μm hypersil silica column (46 × 120 mm) with 480 ml CH $CL_3$, 116 ml methanol and 7 ml 0.5 M $NH_4$ H COO (Ph 3.3) as the mobile buffer. The absorbance of the eluted compound was determined at 280 nm (by spectroflow 773 krakos). The percentage 5-meth dC was calculated as: 5-meth dC = mol 5-meth dC/mol (5-meth dC + dC) × 100%.

### Results

Sixteen patients were treated with Aza-dC. Four out of five patients with resistant leukaemia received Aza-dC as monotherapy and one received Aza-dC in combination with Amsacrine. Only two of these five patients exhibited a slight reduction in leukaemic cells in the bone marrow. Ten out of 11 patients with sensitive leukaemia received Aza-dC in combination with Amsacrine for 2 days and one patient received Aza-dC as monotherapy.

Eight out of this group of 11 patients achieved complete remission (73%), two partial remission; one patient died due to toxicity (Table II). Four patients in CR received one consolidation course consisting of the same schedule (Aza-dC/Amsacrine), except that Aza-dC was given for 4 instead of 6 days. Four out of the eight CR patients relapsed at 11, 8, 7 and 1.5 months, respectively. One patient died 6 weeks after CR, due to a myocardial infarction. Three patients are still in CR at 12, 7 and 2 months, respectively.

In comparison with Ara-C, Aza-dC caused less nausea and vomiting and is therefore better tolerated.

### *Haematological toxicity*

Aza-dC induced a considerable period of myelo-suppression (Table III). A remarkable finding is the phenomenon that the period of a granulocytopenia after the less intensive consolidation course was 10 days longer than after the induction course. Erythro- and mega-karyopoiesis were spared relatively to granulopoiesis. Leukaemic cell reduction in the bone marrow takes much longer than observed in historical controls treated with high dose Ara-C (Figure 1).

### *Non-haematological toxicity*

This consisted of transient sterile peritonitis (severe in three and moderate in three patients) and transient increases in the transaminase and bilirubin levels in one patient. One patient died due to intestinal bleeding; at autopsy a large diverticulum in the jejunum was found (the source of the bleeding). Two patients developed hemiparesis 7 and 10 days after chemotherapy, respectively; a CT-scan revealed no focal abnormalities.

In one case recovery was nearly complete within 2 weeks: in the other it was partial. Three patients experienced a period of somnolence, which began during the course of chemotherapy and persisted until about 3 days after the last Aza-dC infusion.

### *Pharmacokinetic studies*

Aza-dC plasma levels were determined during the 6-h infusion and after discontinuation of the infusion. When Aza-dC was infused at a rate of 21 mg $m^{-2}$ $hour^{-1}$ (125 $mg^{-1}$ $m^{-1}$ 6 $h^{-1}$), the mean plasma concentration was 0.6–1.2 μM. At dose rates of 42 mg $m^{-2}$ $hour^{-1}$ and 84 mg $m^{-2}$ $hour^{-1}$, the plasma levels were about 2 μM and 5 μM, respectively. After discontinuation of the infusion the plasma half-life was 8–14 min.

### *DNA methylation studies* (Table IV)

The methylation level of total DNA in leukaemic bone marrow cells from six patients was measured before (day 0) and after *in vivo* Aza-dC therapy (day 7).

Only bone marrow samples with more than 70% leukaemic cells were used. Because of the slow blast cell reduction, the percentages of leukaemic bone marrow cells on days 0 and 7

**Table II** Results of Aza-dC treatment

| Patient | Outcome | Complications |
|---|---|---|
| *Refractory leukaemia* | | |
| 1 | NR | No |
| 2 | NR | No |
| 3 | NR | No |
| 4 | NR | Reversible hemiparesis; peritonitis; Somnolence |
| 5 | NR | No |
| *Relapsed leukaemia* | | |
| 6 | PR (BM blasts 80%→7%) | Peritonitis; somnolence |
| 7 | CR | Peritonitis; somnolence, hemiparesis |
| 8 | CR | Moderate peritonitis |
| 9 | CR | Moderate peritonitis |
| 10 | CR | No |
| 11 | CR | No |
| 12 | PR (BM blasts 75%→8%) | No |
| 13 | CR | Fever of undetermined origin, hyperbilirubinaemia |
| 14 | Failure | Death due to gastrointestinal bleeding |
| 15 | CR | Moderate peritonitis |
| 16 | CR | No |

**Table III** Haematological recovery from day 1 after chemotherapy, in days

| | *Aza-dC/Amsacrine* | | *Ara-C/Amsacrine* |
|---|---|---|---|
| | *Induction course* $n = 11$ | *Consolidation course* $N = 4$ | *Induction course* $n = 10$ |
| Granulocyte $>0.5 \times 10^{-9}\,l^{-1}$ | 25 (18–29) | 35 (30–30) | 19 (15–22) |
| Reticulocyte >10‰ | 29 (15–24) | 19 (17–21) | 20 (16–22) |
| Platelet transfusion Independent | 18 (16–20) | 16 (13–18) | 19 (14–21) |

Historical controls: Ara-C/Amsacrine: Ara-C 1000 mg m$^{-2}$ × 12; Amsacrine 120 mg m$^{-2}$ × 2 (no. 14).

**Figure 1** Disappearance of leukaemic blast cells from bone marrow after Aza-dC/Amsacrine chemotherapy. Each symbol represents one patient.

were only slightly different in six patients. In all cases there was a decline in DNA methylation levels, which ranged from 15% to 49%. In this small patient group there was no relation between the degree of hypomethylation and the clinical response. For patients 5 and 12, with resistant disease and partial remission, methylation inhibition was 49% and 41%, respectively, whereas patients 10, 11, 13 and 15, who achieved complete remission, showed a methylation inhibition of 15% to 28%. Patient 5, with resistant ALL, exhibited no reduction of leukaemic bone marrow cells on days 6, 14 and 21, whereas methylation decreased to ± 50% on day 6 and returned to the original level on day 21. In two patients tested during leukaemic relapse, methylation was at pretreatment levels.

*Surface marker studies* (Table V)

A complete panel of monoclonal antibodies for cluster differentiation antigens were used to test leukaemic BM cells from seven patients before and 7 days after start of chemotherapy. Because the percentages of leukaemic cells on days 0 and 7 were similar in six cases, a comparison of the cellular immunophenotypes before and after Aza-dC treatment could be made.

Three patients (5, 11 and 16) showed a loss of the early differentiation antigens CD33, and/or CD34. The other three patients did not exhibit significant changes in antigen expression. Patient 5 with resistant ALL lost not only CD34 but also the B cell markers (CD19, CD20 and CD22, although reexpression of these markers occurred on day 21.

For patients 11 and 16 with AML a slight increase in CD11 myeloid differentiation antigens was demonstrated. No change in the other markers (CD's, MPO, TdT, HLA-DR and membrane or cytoplasmic immunoglobulins) was found.

### Discussion

In the present study we demonstrate that Aza-dC is not effective in patients with Ara-C resistant leukaemia. This confirms the observation of cross resistance between Aza-dC and Ara-C in the Ara-C resistant BNML model. Considering the fact that impaired phosphorylation of Ara-C is the underlying mechanism of resistance in the rat leukaemia model (Richel *et al.*, submitted for publication), cross resistance between Ara-C and Aza-dC is not surprising, since both drugs initially follow the same metabolic pathway. In human leukaemia Ara-C resistance is probably a heterogeneous phenomenon, but the lack of antileukaemic activity of Aza-dC in Ara-C refractory patients implies a mechanism involving early intra-cellular metabolic events. The failure of

**Table IV** Methylation levels before (day 0) and after (day 6) in vivo treatment with Aza-dC

| *Patient* | *Day 0* 5-meth dC | *Day 7* 5-meth dC | *Methylation Inhibition* | *Day 21* | *At relapse % 5-meth dC* |
|---|---|---|---|---|---|
| 5 | 4.1 | 2.1 | 49% | 4.1 | – |
| 10 | 4.2 | 3.3 | 22% | | – |
| 11 | 4.8 | 3.5 | 28% | | 4.5 |
| 12 | 4.4 | 2.6 | 41% | | – |
| 13 | 4.9 | 4.2 | 15% | | – |
| 15 | 4.9 | 3.1 | 27% | | 5.0 |

**Table V** Changes in immunophenotype after Aza-dC chemotherapy

| *Patient* | *5* | | *11* | | *16* | |
|---|---|---|---|---|---|---|
| Diagnosis | ALL | | AML | | AML | |
| Clin. response | R | | CR | | CR | |
| % Methylation inhibition | 49 | | 28 | | – | |
| Days | 0 | 7 | 0 | 7 | 0 | 7 |
| % BM blast | 90 | 70 | 90 | 80 | 30 | 40 |
| % Blood dilution | 10 | 15 | 5 | 15 | 10 | 5 |
| % CD34 | 81 | 1 | 0 | 1 | 25 | 2 |
| % CD33 | 0 | 0 | 60 | 1 | 71 | 1 |
| % CD11a | | | 0 | 16 | | |
| % CD11b | | | <1 | 35 | 16 | 47 |
| % CD11c | | | 0 | 14 | | |
| % CD19 | 88 | 13 | | | | |
| % CD20 | 34 | 4 | | | | |
| % CD22 | 71 | 0 | | | | |

Days 0 and 7: before and after start Aza-dC chemotherapy. R: resistant leukaemia, CR: complete remission, CD: cluster differentiation antigen. A complete panel of MoAb was tested, only CDs with significant changes in expression are given.

Aza-dC in patients who had already undergone extensive treatment with Ara-C (Momparler *et al.*, 1985; Debusscher 1989) is probably also due to cross resistance between Ara-C and Aza-dC.

To prove the effectiveness of Aza-dC, as shown in the Ara-C sensitive BNML model (Richel *et al.*, 1988), we selected a group of patients in the first relapse of acute leukaemia after a minimum period of 6 months of unmaintained remission. High-dose Ara-C (HD-Ara-C)/Amsacrine treatment of a similar group AML patients in their first relapse led to CR in approximately 60% of cases (Peters *et al.*, 1988).

We designed a protocol based on administration of Aza-dC as a 6-h infusion twice daily for 6 days. Initially we had chosen for a cumulative Aza-dC dose of 3000 mg $m^{-2}$ during a total infusion time of 72 h. This was based upon the results of clinical studies of Momparler (Momparler *et al.*, 1985), in which the maximal cumulative Aza-dC dose was about 2600 mg $^{-2}$ during a total infusion time of 60 h. Because of neurological and gastrointestinal toxicity the total Aza-dC dose was lowered to 1500 mg $m^{-2}$ during the same infusion time of 72 h. Because Ara-C containing chemotherapy regimens are always combined with an anthracycline or Amsacrine and to ensure the best chance of remission, we added 2 days of Amsacrine to this schedule. We realise that the addition of Amsacrine, although only two doses were given, makes it more difficult to evaluate the contribution of Aza-dC to the response and toxicity of the combined regimen. On the other hand this schedule can be compared to the standard schedule Ara-C/Amsacrine.

Eight out of 11 patients achieved CR (73%). The median duration of remission for these patients was approximately 7 months. Although the patient group is rather small, these data suggest that Aza-dC/Amsacrine is as effective as high-dose Ara-C/Amsacrine (Peters *et al.*, 1988).

The haematological toxicity, as it affected erythro- and megakaryopoiesis, was comparable to that of the high-dose Ara-C/Amsacrine regimen; however, the effect on granulopoiesis was more profound, resulting in agranulopenic periods of 25 and 35 days (Table I) after the induction and consolidation courses, respectively. The differential toxic effects on various haematopoietic cell types is a subject for further research.

A remarkable observation is that the disappearance time for leukaemic blasts in the bone marrow after treatment with Aza-dC/Amsacrine was much longer than that found for historical controls treated with high dose Ara-C/Amsacrine. This phenomenon points to a different mechanism of cytotoxicity compared to that of Ara-C, Aza-dC does not induce inhibition of DNA synthesis, and cytotoxicity only becomes manifest after two cell cycles.

Non-bacterial peritonitis, which became evident about 2–7 days after chemotherapy, was the most frequent non-haematological side-effect. Severe peritonitis were only seen in patients receiving Aza-dC in dosages of 250–500 mg $m^{-2}$. The neurological complications hemiparesis and somnolence were also restricted to patients on these higher dosages. The central nervous system toxicity of Ara-C is of a complete different order and consists mainly of extracerebral toxicity, especially seen at doses higher than 2000 mg $m^{-2}$. In two other clinical studies (Momparler *et al.*, 1986; Debusscher *et al.*, 1989) this kind of toxicity was not encountered. These complications are probably related to the high Axa-dC plasma levels. In Momparler's study plasma levels of 0.8–1.4 $\mu$M were obtained for 60 h, while in our study 250–500 mg $m^{-2}$ Aza-dC resulted in plasma levels of 1.8–5 $\mu$M for 72 h. A dosage of 125 mg $m^{-2}$ Aza-dC yielded plasma levels of 0.6–1.2 $\mu$M. Although only a limited number of patients has been given this lower dose, toxicity seems to be reduced. In the small group of patients tested for DNA methylation no association existed between the degree of hypomethylation and clinical response.

Although the loss of the stem cell marker CD34 and the early myeloid progenitor marker CD33 in three patients after Aza-dC is suggestive of leukaemic cell differentiation, further research is needed to elucidate this interesting phenomenon, especially with regard to the increased expression of markers representing more mature phenotypes.

The data in this report demonstrate that Aza-dC combined with Amsacrine according to the schedule described above has a major antileukaemic effect in patients who have not previously undergone extensive Ara-C therapy. This combination is probably as effective as HD-Ara-C/Amsacrine.

Drug-related toxicity, especially at the higher dosages, was considerable but generally reversible. When an Aza-dC dose of 125 mg $m^{-2}$ was administered 12 times as a 6 h infusion toxicity was acceptable.

More experience with Aza-dC for remission and/or consolidation therapy is needed to find out whether this drug will gain a place as therapy-of-choice for acute leukaemia.

### References

BOUCHARD, J. & MOMPARLER, R.L. (1983). Incorporation of 5-Aza-2′-Deoxycytidine-5′-Triphosphate into DNA. *Molecular Pharmacol.*, **24**, 109.

COLLY, L.P., RICHEL, D.J., ARENTSEN-HONDERS, W., STARRENBURG, C.W.J., EDELBROEK, P.M. & WILLEMZE, R. (1991). A simplified assay for measurement of cytosine-arabinoside incorporation into DNA in Ara-C sensitive and resistant leukemia cells. *Pharmacokinetics*, (in press).

CREUSOT, F., ACS, G. & CHRISTMAN, J.K. (1982). Inhibition of DNA methyl-transferase and induction of Fried erythroleukemia cell differentiation by 50 Azacytidine and 5-Aza-2′- deoxycytidine. *J. Biol. Chem.*, **257**, 2041.

DAVIS, R.W., THOMAS, M., CAMERON, J., ST. JOHN, T.P., SCHERER, S. & PADGET, R.A. (1980). Rapid DNA isolation for enzymatic and hybridization analysis. *Methods Enzymol.*, **65**, 404.

DEBUSSCHER, L., MAIRIE, J.P., DOCHIN, P. & 4 others (1989). Phase I-III trial of 5-Aza-2′-deoxycytidine in adult patients with acute leukemia. *Proceedings: 5 Aza-2-deoxycytidine, Preclinical and Clinical Studies*. Symposium, Amsterdam 1989. ISBN 90-72973-01-1.

LEY, T.J., DESIMONE, J., ANAGNON, N.P., KELLER, G.H. & others (1982). 5-Azacytidine selectively increases gammaglobulin synthesis in a patient with $\beta^+$ thalassemia. *N. Engl J. Med.*, **37**, 1469.

LIN, K.-T., MOMPARLER, R.L. & RIVARD, G.E. (1985). Sample preparation for the determination of 5-aza-2′-deoxycytidine in plasma by high performance liquid chromatography. *J. Chromatog.*, **345**, 162.

MOMPARLER, R.L. & GONZALES, F.A. (1978). Effect of intravenous infusion of 5-Aza-2′-deoxycytidine on survival time of mice with L1210 leukemia. *Cancer Res.*, **38**, 2673.

MOMPARLER, R.L., RIVARD, G.E. & GYGER, M. (1985). Clinical trial of 5-Aza-2 deoxycytidine in patients with acute leukemia. *Pharmacol. & Therap.*, **30**, 277.

PALLESEN, J.G. & PLESNEN, T. (1987). The Third International Workshop and Conference on human leukocyte differentiation antigen with an up to date overview of the CD nomenclature. *Leukemia*, **1**, 231.

PETERS, W.G., WILLEMZE, R. & COLLY, L.P. (1988). Results of induction and consolidation treatment with intermediate and high-dose cytosine arabinoside and m-Amsa of patients with poor risk acute myelogenous leukemia. *European J. Haematol.*, **40**, 198.

RICHEL, D.J., COLLY, L.P., ARKESTEIJN, G.J.A., ARENTSEN-HONDERS, M.W., TER RIET, P.M. & WILLEMZE, R. Substrate specific deoxycytidine kinase deficiency in Ara-C resistant leukemic cells. (Submitted).

RICHEL, D.J., COLLY, L.P., LURVINK, E. & WILLEMZE, R. (1988). Comparison of the anti-leukaemic activity of 5-Aza-2-deoxycytidine and arabinofuranosyl-cytosine in rats with myelocytic leukaemia. *Br. J. Cancer*, **58**, 730.

VESELY, J. & CIHAK, A. (1977). Incorporation of a potent antileukemic agent, 5-Aza-2′-deoxycytidine, into DNA of cells from leukemic mice. *Cancer Res.*, **37**, 3684.

WILSON, V.L., JONES, P.A. & MOMPARLER, R.L. (1983). Inhibition of DNA methylation in L1210 leukemic cells by 5-Aza-2′-deoxycytidine as a possible mechanism of chemotherapeutic action. *Cancer Res.*, **43**, 3493.